# Fulminant Pulmonary Hypertension Due to Pulmonary Lymphangitic Carcinomatosis and Tumor Embolism From Occult Pulmonary Adenocarcinoma

**DOI:** 10.7759/cureus.111909

**Published:** 2026-07-01

**Authors:** Sai Shahane, Sankalp Acharya, Rutuja Challawar, Jillianne Unas, Patricia Marie Perez de Tagle, Doantrang Du, Sharon Weiner

**Affiliations:** 1 Internal Medicine, Monmouth Medical Center, Long Branch, USA; 2 Internal Medicine, RWJBarnabas Health, Long Branch, USA; 3 Pulmonology, Rutgers Health, Monmouth Medical Center, Long Branch, USA

**Keywords:** pulmonary adenocarcinoma, pulmonary hypertension, pulmonary lymphangitic carcinomatosis, pulmonary tumor embolism, right ventricular failure

## Abstract

Pulmonary lymphangitic carcinomatosis (PLC) and pulmonary tumor embolism (PTE) are rare manifestations of metastatic malignancy that may mimic more common causes of dyspnea and pulmonary hypertension, often leading to delayed diagnosis. Both conditions are associated with poor prognosis and may present with rapidly progressive right ventricular failure. A 45-year-old female lifelong non-smoker with no significant past medical history presented with a two-week history of cough, pleuritic chest pain, and worsening fatigue. CT pulmonary angiography was negative for central macrovascular pulmonary embolism but demonstrated interlobular septal thickening, ground-glass opacities, pulmonary nodules, and vertebral lesions. Initial echocardiography showed mild to moderate pulmonary hypertension with preserved right ventricular function. Despite supportive management, the patient experienced rapid clinical deterioration, progressing to severe pulmonary hypertension, right ventricular failure, cardiogenic shock, and pulseless electrical activity arrest. Post-mortem examination revealed invasive pulmonary adenocarcinoma with extensive lymphovascular invasion and metastatic disease, consistent with PLC and PTE. This case emphasizes the need to take into account PLC and PTE in patients with rapidly progressive pulmonary hypertension despite negative pulmonary embolism imaging. Recognition of subtle radiographic clues may facilitate earlier diagnosis, although prognosis remains poor once right ventricular failure develops.

## Introduction

Pulmonary lymphangitic carcinomatosis (PLC) and pulmonary tumor embolism (PTE) are rare manifestations of metastatic malignancy that may mimic more common causes of dyspnea, including pulmonary embolism. Both conditions can present with progressive respiratory symptoms, pulmonary hypertension, and right ventricular strain; however, imaging findings are often nonspecific, and diagnosis is frequently delayed or established post-mortem [1-3].

We report a case of occult pulmonary adenocarcinoma presenting with rapidly progressive pulmonary hypertension and acute right ventricular failure, highlighting the diagnostic challenges of malignant pulmonary vascular and lymphatic involvement.

## Case presentation

A 45-year-old female lifelong non-smoker with no significant past medical history presented with a two-week history of non-productive cough, pleuritic left-sided chest pain, and worsening fatigue. On presentation, she was hemodynamically stable (oxygen saturation 92% on room air, pulse 90 beats/min, blood pressure 122/62 mmHg, and temperature 98.1°F). ECG was unremarkable, and serum troponin was normal. Laboratory investigations revealed a C-reactive protein level of 7.3 mg/dL (reference range: <7.0 mg/L), D-dimer of 7.2 mg/L (reference range <0.5 mg/L), and B-type natriuretic peptide (BNP) of 47 pg/mL (reference range: <100 pg/mL).

CT pulmonary angiography (CTPA) showed no evidence of pulmonary embolism; however, it demonstrated mild dilation of the main pulmonary artery, bilateral interlobular septal thickening, patchy ground-glass opacities, multiple nonspecific pulmonary nodules, and lucencies in the T2 and T3 vertebral bodies (Figures 1-2). Transthoracic echocardiography revealed a preserved left ventricular ejection fraction (55-60%), mildly elevated right ventricular systolic pressure (RVSP) of 46 mmHg, and normal right ventricular size and function, consistent with mild to moderate pulmonary hypertension. Further infectious and autoimmune workup was unremarkable. A CT of the abdomen and pelvis showed no evidence of a primary intra-abdominal malignancy, and tumor markers, including carcinoembryonic antigen, CA 19-9, and CA-125, were negative. Mammography was also normal. Given the vertebral lesions, a bone biopsy was performed.

**Figure 1:**
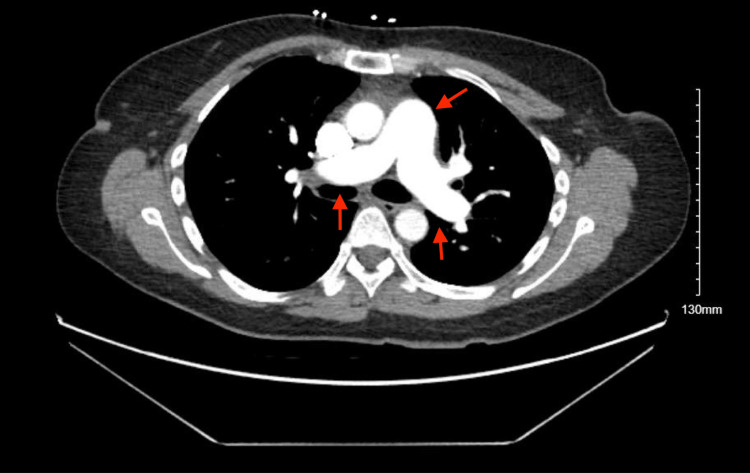
CT pulmonary angiogram negative for acute pulmonary embolism CT pulmonary angiogram showing no intraluminal filling defects within the pulmonary trunk or main pulmonary arteries, excluding acute pulmonary embolism as the cause of the patient's pulmonary hypertension and right ventricular dysfunction. CT: computed tomography

**Figure 2:**
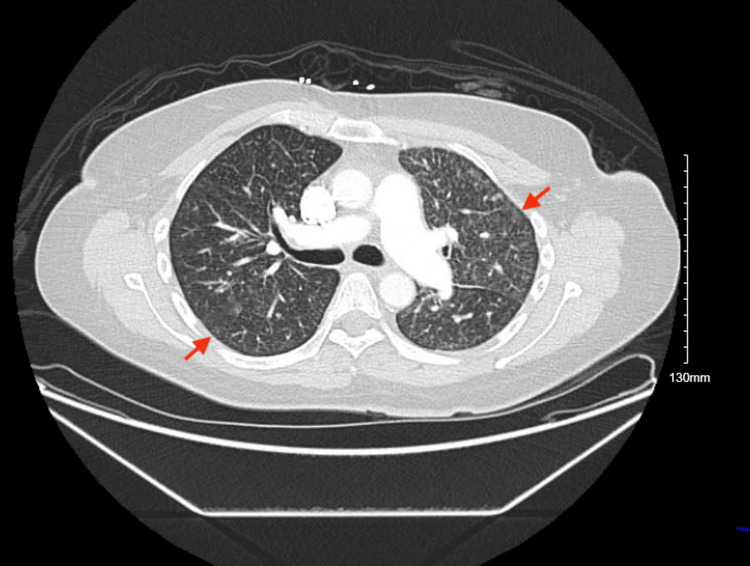
CT chest showing diffuse bilateral pulmonary parenchymal abnormalities Axial contrast-enhanced CT image of the chest demonstrating bilateral nodular interlobular septal thickening (arrows), a characteristic radiologic feature of PLC, along with patchy ground-glass opacities and scattered pulmonary nodules. CT: computed tomography, PLC: pulmonary lymphangitic carcinomatosis

Two days after hospital admission, the patient experienced acute clinical deterioration, with worsening dyspnea, tachypnea, tachycardia, and hypotension. ECG demonstrated right axis deviation and T-wave inversions. Repeat echocardiography revealed severe pulmonary hypertension with RVSP of 79 mmHg, right ventricular dilation, and reduced right ventricular systolic function. This represented a rapid increase in RVSP from 46 mmHg to 79 mmHg within 48 hours, highlighting the fulminant progression of pulmonary hypertension. BNP increased to 1500 (reference range: <100 pg/mL), and arterial blood gas analysis showed metabolic acidosis. Bone biopsy pathology was pending at the time of the patient’s clinical deterioration. She required intubation and vasopressor support.

Despite aggressive management, the patient developed pulseless electrical activity arrest within a few hours and died. Post-mortem examination revealed invasive pulmonary adenocarcinoma with extensive lymphovascular invasion and metastases to mediastinal lymph nodes, thyroid, spine, and epicardium, consistent with PLC and PTE.

## Discussion

PLC and PTE are rare manifestations of metastatic malignancy characterized by dissemination of tumor cells within the pulmonary lymphatic and vascular systems, respectively. These conditions frequently coexist and represent a spectrum of tumor-related pulmonary involvement [1]. PLC accounts for approximately 6-8% of pulmonary metastases and is most commonly associated with adenocarcinomas, particularly of the lung, breast, and gastrointestinal tract [1]. Pathogenesis involves lymphatic obstruction leading to interstitial edema and interlobular septal thickening. PLC typically occurs in patients with established malignancy and is rarely the initial presentation of occult cancer [1]. Unlike the typical presentation in patients with known malignancy, PLC and PTE were the initial manifestations of occult pulmonary adenocarcinoma in our patient.

Patients typically present with progressive exertional dyspnea, dry cough, weight loss, hypoxia, and evolving right ventricular dysfunction over weeks to months [2]. Our patient’s pleuritic chest pain was likely related to invasion of the subpleural lymphatics.

Imaging findings in PLC and PTE are often nonspecific. They may mimic more common conditions such as pulmonary thromboembolism, pneumonia, or interstitial lung disease, frequently contributing to delayed diagnosis and advanced disease at presentation. Common CT findings include interlobular septal thickening, ground-glass opacities, pulmonary nodules, hilar lymphadenopathy, and pleural effusions [3]. A study of 35 patients with PLC identified nodular interlobular septal thickening as the most frequent CT abnormality [4]. In our patient, these radiographic findings in conjunction with rapidly progressive pulmonary hypertension despite negative pulmonary embolism imaging suggested diffuse malignant infiltration of the pulmonary lymphatic and vascular systems. Although CTPA excluded central macrovascular pulmonary thromboembolism, it cannot detect microscopic pulmonary tumor emboli within the pulmonary microvasculature, which likely accounted for the rapid progression of pulmonary hypertension despite negative pulmonary embolism imaging. Elevated D-dimer may also occur in tumor-associated pulmonary vascular disease, potentially contributing to diagnostic uncertainty [2]. Ventilation-perfusion imaging may be normal in PLC; however, a characteristic lymphangitic pattern (“contour mapping pattern” of multiple small linear peripheral perfusion defects) has been associated with poor prognosis [5]. PET/CT may also aid diagnosis, with reported sensitivity and specificity of 86% and 100%, respectively, for detecting PLC [4]. Serial echocardiography may also aid in identifying rapidly progressive pulmonary hypertension and evolving right ventricular dysfunction.

Definitive diagnosis frequently requires invasive procedures such as transbronchial biopsy or pulmonary artery sampling, which may not be feasible in critically ill patients; thus, many cases are diagnosed post-mortem [3]. Management of PLC and PTE is challenging and largely directed toward treatment of the underlying malignancy when feasible. Supportive measures may include oxygen therapy, mechanical ventilation, vasopressor support, and management of right ventricular failure. Although pulmonary vasodilator therapies have been attempted in selected cases of tumor-associated pulmonary hypertension, evidence remains limited, and outcomes are generally poor [2,3]. In the presence of pulmonary venous obstruction, pulmonary vasodilators may precipitate pulmonary edema by increasing pulmonary artery blood flow against elevated post-capillary venous resistance or worsen hypoxemia through ventilation-perfusion mismatch [6]. Corticosteroids have also been used empirically in some cases of PLC to reduce inflammatory edema and improve symptoms, although evidence supporting their efficacy remains limited [3]. Early recognition is important because prompt initiation of systemic chemotherapy in selected patients may occasionally improve symptoms or pulmonary hemodynamics; however, most patients present with advanced disease that precludes definitive oncologic therapy [2,3]. In our patient, the fulminant clinical deterioration and rapidly progressive right ventricular failure precluded cancer-directed treatment prior to diagnosis.

A systematic review of 107 reported cases of PLC demonstrated a mean survival of approximately 158 days following symptom onset [7]. In contrast, an autopsy series of 20 patients with neoplastic pulmonary emboli reported a mean survival of approximately one month [8]. Consistent with this aggressive disease spectrum, our patient experienced rapid deterioration, progressing from initial symptom onset to death within three weeks.

## Conclusions

PLC and PTE are rare but important causes of rapidly progressive pulmonary hypertension and acute right ventricular failure. These entities should be considered in patients with unexplained pulmonary hypertension, particularly when CT pulmonary angiography is negative for central macrovascular pulmonary thromboembolism despite rapidly progressive pulmonary hypertension and right ventricular strain, and imaging demonstrates nodular or irregular interlobular septal thickening, ground-glass opacities, pulmonary nodules, or other features suggestive of an infiltrative process. Because clinical and radiographic findings are often nonspecific, diagnosis remains challenging and is frequently delayed.

This case highlights how malignant pulmonary vascular and lymphatic involvement may represent the first manifestation of an occult malignancy and can lead to fulminant clinical deterioration. Serial echocardiography demonstrating rapidly worsening pulmonary pressures and evolving right ventricular dysfunction may provide an important clue to the diagnosis.
